# Chitosan oligosaccharide ameliorates acute lung injury induced by blast injury through the DDAH1/ADMA pathway

**DOI:** 10.1371/journal.pone.0192135

**Published:** 2018-02-07

**Authors:** Yun-En Liu, Cang-Ci Tong, Yu-Biao Zhang, Pei-Fang Cong, Xiu-Yun Shi, Ying Liu, Lin Shi, Zhou Tong, Hong-Xu Jin, Ming-Xiao Hou

**Affiliations:** Emergency Medicine Department of General Hospital of Shenyang Military Command, Laboratory of Rescue Center of Severe Wound and Trauma, PLA, Shenhe District, Shenyang, China; Brown University, UNITED STATES

## Abstract

**Objective:**

To investigate the protective effect of chitosan oligosaccharide (COS) on acute lung injury (ALI) caused by blast injury, and explore possible molecular mechanisms.

**Methods:**

A mouse model of blast injury-induced ALI was established using a self-made explosive device. Thirty mice were randomly assigned to control, ALI and ALI + COS groups. An eight-channel physiological monitor was used to determine the mouse physiological index. Enzyme linked immunosorbent assay was used to measure serum inflammatory factors. Hematoxylin-eosin staining, terminal deoxynucleotidyl transferase dUTP nick end labeling assay, immunofluorescence staining, real time-polymerase chain reaction and western blot assay were used to detect inflammatory reactions, oxidative stress and apoptosis.

**Results:**

Mice were sacrificed 24 hours after successful model induction. Compared with the ALI group, the heart rate, respiration and PCO_2_ were significantly lower, but the PO_2_, TCO_2_ and HCO_3_^-^ were significantly higher in the ALI + COS group. Compared to ALI alone, COS treatment of ALI caused a significant decrease in the wet/dry lung weight ratio, indicating a reduction in lung edema, inflammatory cell infiltration, levels of tumor necrosis factor-α, interleukin (IL)-1β, IL-4, IL-6 and nuclear factor kappa B mRNA and protein expression were reduced and IL-10 mRNA and protein expression was increased (*P* < 0.05). COS significantly inhibited reactive oxygen species, MDA5 and IREα mRNA and protein expressions, cell apoptosis and Bax and Caspase-3 mRNA and protein expressions, and significantly increased superoxide dismutase-1 mRNA expression, and Bcl-2 and Caspase-8 mRNA and protein expression (all *P*<0.05). COS significantly increased dimethylarginine dimethylaminohydrolase 1 (DDAH1) protein expression, and reduced ADMA and p38 protein expression (*P*< 0.05).

**Conclusion:**

Blast injury causes inflammation, oxidative stress and apoptosis in the lung tissues of mice. COS has protective effects on blast injury-induced ALI, possibly by promoting DDAH1 expression and inhibiting ADMA and mitogen-activated protein kinase pathways.

## Introduction

Blast injury is a complex form of physical damage caused by the direct or indirect action of a blast wave, commonly found in terrorist incidents, and gas and underground explosions[1]. Blast injury is characterized by mild trauma, severe internal injuries, complex injuries, high shock rate, and high mortality. The lung is one of the most frequently affected organs and lung injury is one of the causes of high mortality[2]. The shock wave caused by the explosion acts on the body, causing the deformation of the chest and increased intrathoracic pressure. Through lung parenchymal transmission, the shock wave leads to immediate or delayed massive hemorrhage, and alveolar and pulmonary capillary rupture that cause pulmonary contusion, pulmonary hemorrhage and pulmonary edema[3]. Worsening conditions can cause acute lung injury (ALI), acute respiratory distress syndrome and multiple organ dysfunction syndrome[4]. ALI commonly accompanies inflammation, reactive oxygen species release, glutamate toxicity and mitochondrial dysfunction[5]. Therefore, it is of great importance to study the mechanism and treatment of pulmonary blast injury for treatment of wounded individuals.

ADMA is an inhibitor of endogenous nitric oxide synthase, thus reducing the production of nitric oxide, leading to vascular endothelial injury, and aggravatation of inflammatory reactions[6]. ADMA is strongly associated with hypertension, diabetes and pulmonary hypertension[7–9]. ADMA also increases the generation of oxygen free radicals, activates the nuclear factor kappa B (NF-кB) pathway, and is involved in the regulation of inflammatory reactions[10]. ADMA levels in serum can be used as an indicator of inflammation and oxidative stress[11]. Advanced glycation end products and advanced inflammatory mediator high mobility group box protein 1 (HMGB1) levels are independent of the ADMA level in patients[12]. Dimethylarginine dimethylaminohydrolase 1 (DDAH1) is a major hydrolase of ADMA, forming citrulline and dimethylamine, that are excreted by the kidney[13]. A previous study has shown that overexpression of the DDAH1 gene reduces inflammatory reactions by decreasing ADMA expression[14]. DDAH1 was reported to increase nitric oxide levels *in vivo* by activating the Ras/PI3K/Akt pathway[15]. Thus, we hypothesized that DDAH1/ADMA axis is protective in ALI induced by blast injury.

Chitosan oligosaccharide (COS) has good water solubility, is easy to absorb and use, inhibits tumor growth and inflammatory reactions, enhances bone strength, and provides resistance against bacteria, malaria, andfungi[16]. COS also accelerates wound healing, activates B lymphocytes and T lymphocytes[17, 18], enhances cellular and humoral immune functions, activates the tumor killing activity of macrophages and promotes interleukin (IL)-1 formation[19]. However, the protective effect of COS on blast injury-induced ALI and its molecular mechanism of regulating ALI remain poorly understood. This study established mouse models of blast injury-induced ALI using a self-made explosive device, explored the protective effect of COS on blast injury, and investigated its possible mechanism of action.

## Materials and methods

### Materials

https://www.protocols.io/view/untitled-protocol-k2ncyde.

#### Experimental animals

Thirty healthy male Kunming mice weighing 2530 g and aged 6–8 weeks were purchased from the Experimental Animal Center of the General Hospital of Shenyang Military in China. All mice were housed in the Experimental Animal Center of the General Hospital of Shenyang Military, and allowed free access to food and water. Animal welfare and experimental design were approved by the Ethics Committee of the General Hospital of Shenyang Military.

#### COS

COS was gifted by the Institute of Metal Research, Chinese Academy of Sciences, China. The degree of polymerization of COS is 2–15, with a mean molecular weight of 100 kDa and 90% deacetylation.

### Methods

#### Establishment of blast injury-induced ALI in mice

Mouse models of blast injury-induced ALI were established using a self-made explosive device designed as follows: the lung blasting simulation device consists of four parts: air compression device, fixture, protection device and data acquisition device. The bottom of the device is the air compression device, a length of about 100 cm of steel pipe connected to the air pressure pump and power supply. The main device is above a 30 cm steel pipe, the top surface for the wire. The fixed protective cover contains the middle of the connection pressure sensor. The top of the main body and the lower device can be placed in the middle of different thicknesses of an aluminum film attached by screws. The shock wave can be increased by increasing the aluminum thickness. The protection device consists of a hard plastic cylinder, containing a hole for mice to enter, allowing specific parts of the mouse to be exposed to the shock wave. The instantaneous pressure and duration of the shock wave were recorded using PCB pressure sensor (PCB, GE Company, USA), amplifier, signal receiver and data acquisition system software. The instantaneous shock wave overpressure was 321±24 PSI.

#### Experimental protocol

After acclimation, thirty mice were divided randomly into three groups: 1) control group; 2)ALI group and 3)ALI + COS group. At 14 days before blast injury, mice in the ALI + COS group were administered COS 80 mg/kg per day by gavage. While mice in the control group and ALI group were given the same volume of distilled water.

#### Detection of physiologic parameters

At 24 hours after blast injury, mice were intraperitoneally anesthetized with pentobarbital. A longitudinal incision was made on the neck. After muscles were isolated, the trachea was exposed and carefully dissociated. A “T”-shape incision was made with eye scissors at the site of two tracheal rings below the cricoid cartilage. A “Y”-shape endotracheal tube was inserted after being connected to an eight-channel physiological monitor (MPA2000; GE Company, USA) to record pulmonary function. Lung function parameters were calculated as esophageal pressure, airway pressure and gas flow. After left femoral artery catheterization and connecting a heart function analyzer and blood pressure sensor, the three-lead electrocardiogram and mouse heart rate was continuously monitored. Body temperature, heart rate, respiratory rate, partial pressure of carbon dioxide (PCO_2_), oxygen partial pressure (PO_2_), pH, alveolar-arterial oxygen partial pressure difference (AaDO_2_), total amount of carbon dioxide (TCO_2_) and bicarbonate ion (HCO_3_^-^) were detected.

#### Sample collection

After 12 hours of fasting and 4 hours of water deprivation preoperatively, mice were intraperitoneally anesthetized with 2% sodium pentobarbital (1.5 ml/kg), and fixed on the operating table in a prone position. The abdominal cavity was opened and blood was harvested through the abdominal aorta. Serum was isolated by centrifugation, and serum factors were examined using enzyme linked immunosorbent assay (ELISA). The left lung was immersed in 10% formalin buffer, embedded in paraffin, and sliced into 3-4mm sections. These sections were stained with hematoxylin and eosin. The surface of the upper lobe of the right lung was dried with filter paper. After weighing on an analytical balance, the wet weight was recorded. The right lung was dried in a 60°C oven for 72 hours, and its dry weight was recorded. The ratio of dry weight to wet weight was calculated which changes in the opposite direction of edema formation. The remaining lung was placed in a nitrogen canister for mRNA and protein determination.

#### Enzyme linked immunosorbent assay (ELISA)

ELISA kits (Cloud-Clone Company, USA) were utilized to measure serum TNF-α, IL-1β, IL-4, IL-6 and IL-10 levels, following the manufacturer’s instructions. A 100-μl aliquot of standard solution or 100 μl of diluted sample was added to reaction plates, mixed and then incubated at 37°C for 30 min. After the plate was washed, 100-μl detection solution (with the primary antibody) was added to each well and plates were incubated at 37°C for 2 h. After the plate was washed, 100-μl HRP-labeled secondary antibody was added to each well and plates were incubated at 37°C for 30 min. After the plate was washed, 50 μl of staining solution A and 50 μl of staining solution B were added, with incubation for 15 min in the dark. A 50-μl aliquot of stop buffer was added to terminate the reactions. Optical density values were measured at 450 nm with a microplate reader (Bio-Rad, USA). Standard curves were used to calculate concentrations in each sample.

#### Western blot assay

The protein sample was added to the corresponding sodium dodecyl sulfate (SDS) gel sample buffer, boiled, denaturalized for 5 minutes, before SDS-polyacrylamide gel electrophoresis, and then transferred onto membranes. The membranes were blocked in phosphate buffered saline with Tween (PBST) containing 5% defatted milk powder at room temperature for 1 hour, washed three times with PBST, and incubated with primary antibodies for NF-κB, TNF-α, IL-1β, IL-4, IL-6, IL-10, SOD-1, MDA5, IREa, Bcl-2, Bax, Caspase3, Caspase8, DDAH1, ADMA and p38(Sigma, USA) overnight at 4°C. Subsequently, the blot was washed and incubated with goat antimouse IgG conjugated to peroxidase(Sigma, USA). Antibody binding was detected by chemiluminescence staining using an ECL detected kit (Bio-Rad, USA). The density of each band was quantified by densitometry using Bandscan 5.0 software.

#### Real time-PCR

Total RNA was extracted using Trizol reagent (Invitrogen, USA). Real time-PCR was conducted using SYBR Premix Ex Taq (TaKaRa, Japan) and an Mx3000P instrument (Sigma, USA). mRNA expression was analyzed using Stratagene Mx3000P software. The mRNA expression of target genes was normalized to a control glyceraldehyde-3-phosphate dehydrogenase (GAPDH) using the comparative threshold cycle method.

#### Immunofluorescence staining

The lung was dewaxed with xylene, hydrated with a graded alcohol series, incubated with 0.1% Triton X-100 for 30 minutes, and washed three times with PBS for 5 minutes each time. Samples were blocked with 5% bovine serum albumin and 10% goat serum, each for 30 minutes, incubated with primary antibody overnight at 4°C in a wet box, and stained with a fluorescent secondary antibody. Finally, samples were observed and photographed with a microscope.

#### Reactive oxygen species (ROS) determination

Frozen sections of the lung were immersed in PBS twice for 5 minutes each time, incubated with 10 μM dihydroethidium in the dark for 20 minutes, immersed in PBS twice for 5 minutes each time, and observed and photographed using a fluorescence microscope.

#### TUNEL staining

Changes in apoptotic cells were detected in accordance with the instruction of the TUNEL kit (Cloud-Clone Company, USA). Stained with a brown nucleus were deemed apoptotic cells. Five different fields were randomly selected under a high-power microscope (×400) to calculate the apoptotic index.

#### Statistical analysis

Data were expressed as the mean ± standard deviation, and analyzed using SPSS 20.0 software. Measurement data were analyzed using the t-test and analysis of variance. All statistical tests were two-tailed probability tests. Statistical significance was set to α = 0.05.

## Results

### COS attenuates blast injury-induced physiologic parameters

**Fig 1:**
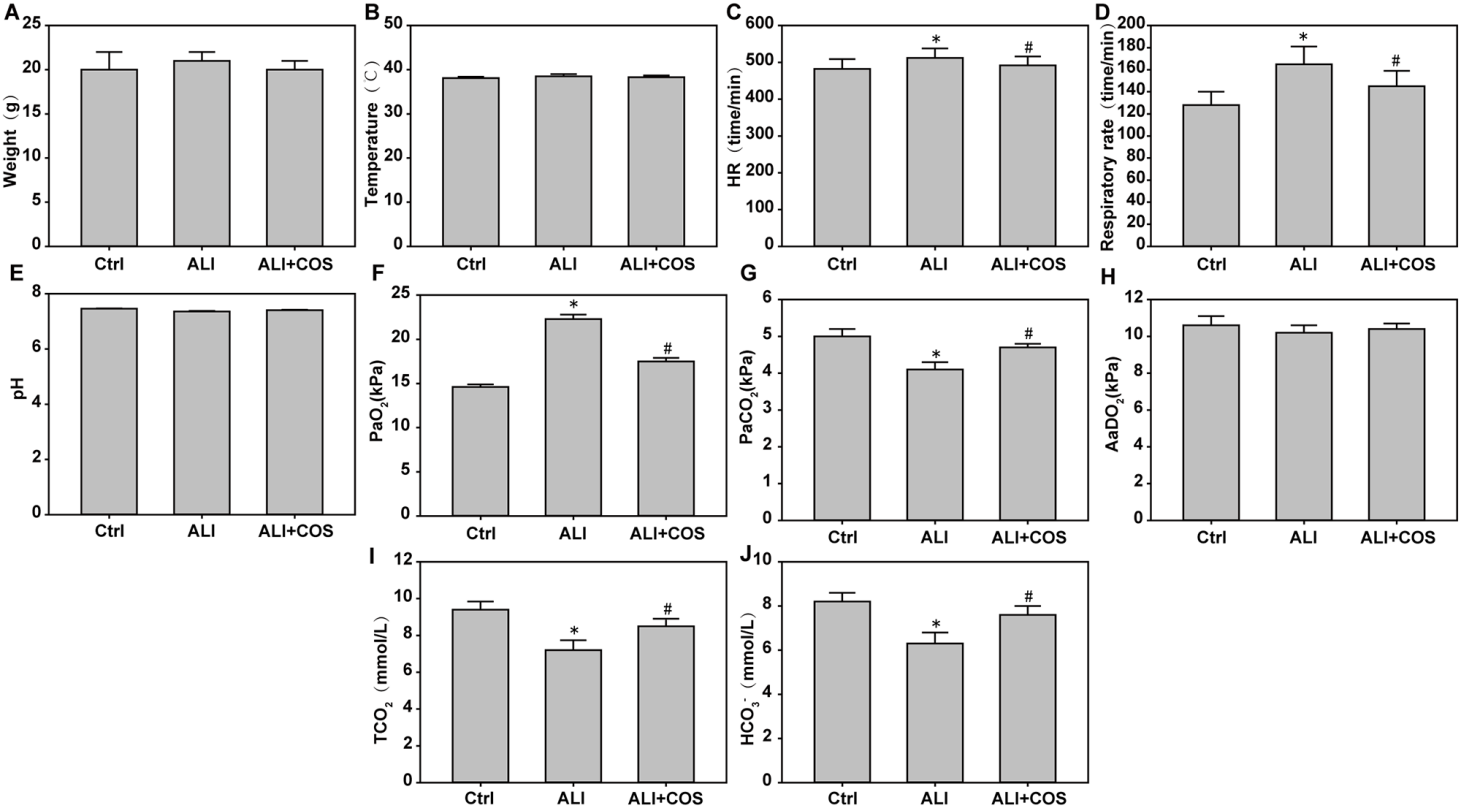
COS effects on mouse weight, heart rate, respiratory rate and blood gas analysis. The MPA2000 eight-channel physiological monitor was used to record pulmonary function. Lung function parameters were calculated by esophageal pressure, airway pressure and gas flow. After left femoral artery catheterization, connecting heart function analyzer and blood pressure sensor, mouse heart rate was continuously monitored. *P < 0.05, vs. control group; ^#^*P* < 0.05, vs. the ALI group. COS: chitosan oligosaccharide; ALI: acute lung injury.

Mice were sacrificed 24 hours after blast injury. theheart rate, respiratory rate and PaO_2_ were increased significantlyand the PaCO_2_, TCO_2_ and HCO_3_^-^ were decreased significantly in the ALI group compared with the control group (Fig 1A–1F, *P* < 0.05). The heart rate, respiratory rate and the PaO_2_ were decreased significantly, and PaCO_2_, TCO_2_ and HCO_3_^-^ were increased significantly in the ALI + COS group compared with the ALI group (Fig 1A–1F, *P* < 0.05).

### COS ameliorats blast injury-induced wet/dry lung weight ratio and the degree of injury

**Fig 2:**
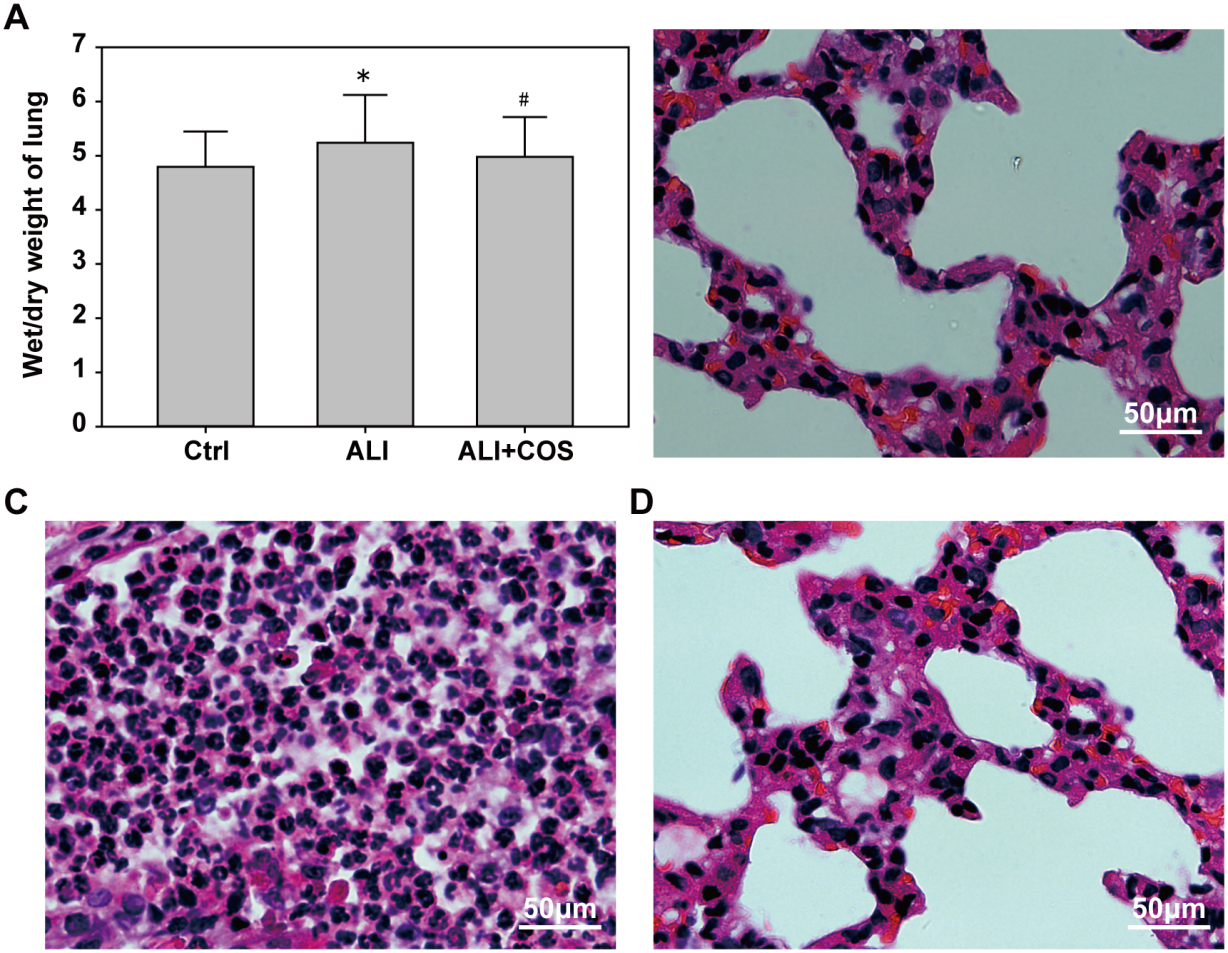
COS effects on the wet/dry weight ratio and pathological changes in the lung of mice. *P < 0.05, vs. control group; ^#^P < 0.05, vs. ALI group. COS: chitosan oligosaccharide; ALI: acute lung injury.

Compared with the control group, the wet/dry lung weight ratio was significantly increased in the ALI group, and COS restored the wet/dry lung weight ratio to normal levels (Fig 2A, *P* < 0.05). Compared with the ALI group, COS markedly inhibited the blast injury-induced infiltration of inflammatory cells, and reduced alveolar wall thickening (Fig 2B–2D).

### COS reduces blast injury-induced serum inflammatory factor expression in mice

**Fig 3:**
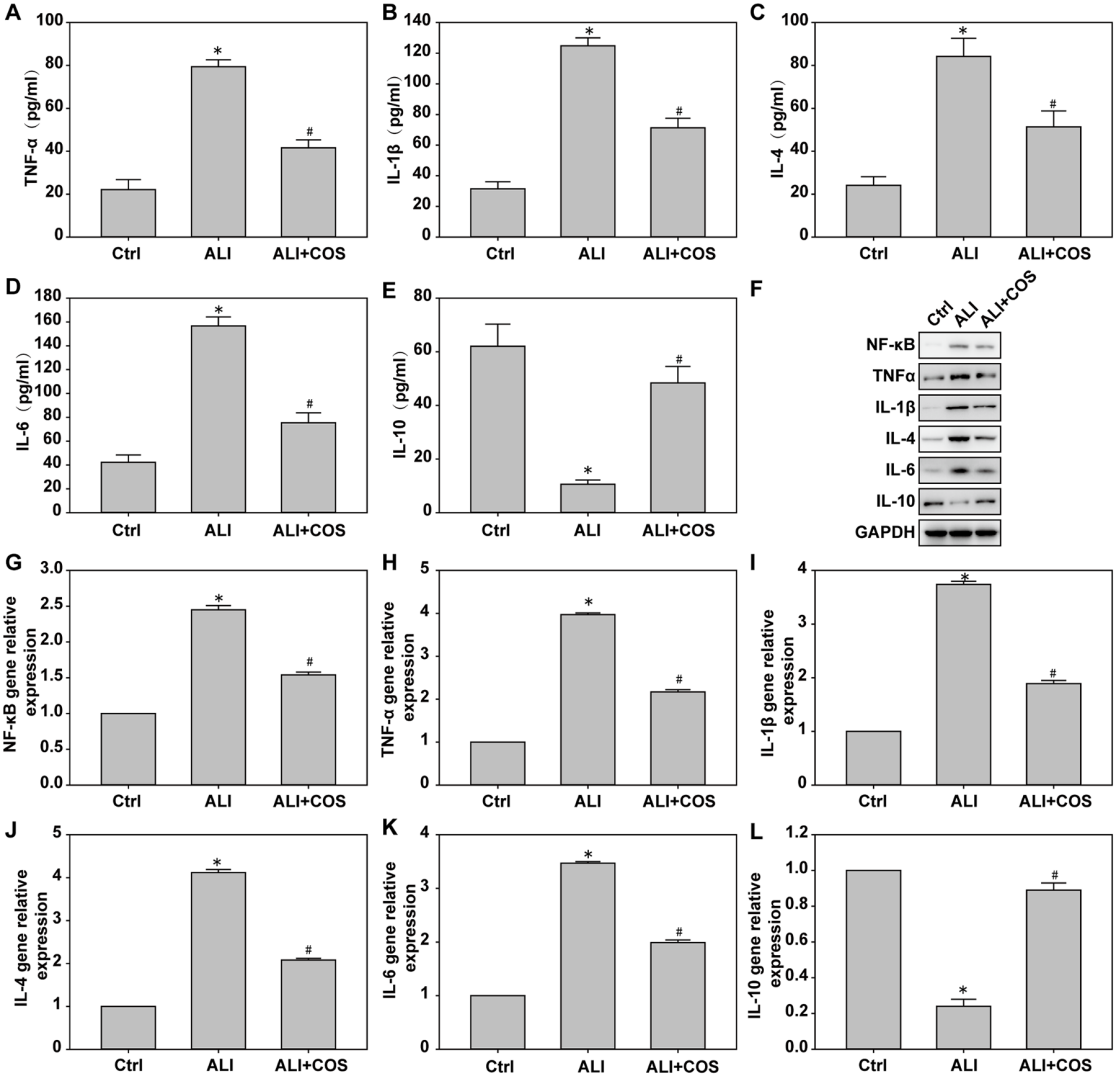
COS effects on inflammatory factor expression in the lung. The expression of TNF-α, IL-1β, IL-4, IL-6 and IL-10 in serum were determined using ELISA kit. The expression of inflammatory factors was detected using real time-PCR and western blot. *P < 0.05, vs. control group; ^#^*P* < 0.05, vs. ALI group. COS: chitosan oligosaccharide; ALI: acute lung injury; TNF: tumor necrosis factor; IL: interleukin.

Compared with the control group, the expression of serum TNF-α, IL-1β, IL-4 IL-6 and NF-κB were significantly increased, and IL-10 expression was significantly decreased in the ALI group. Compared with the ALI group, the expressions of serum TNF-α, IL-1β, IL-4 and IL-6 were significantly decreased, and IL-10 expression was significantly increased in the ALI + COS group (Fig 3A–3E, *P* < 0.05).

### COS suppresses blast injury-induced lung inflammatory factor expression in mice

Compared with the control group, lung tissue levels of NF-κB, TNF-α, IL-1β, IL-4 and IL-6 mRNA and protein were significantly increased, and IL-10 mRNA and protein expression was significantly decreased in the ALI group. Compared with the ALL group, levels of NF-κB, TNF-α, IL-1β, IL-4 and IL-6 mRNA and protein were significantly decreased, and IL-10 mRNA and protein expression was significantly increased in the ALI + COS group (Fig 3F–3L, P < 0.05).

### COS decreases blast injury-induced ROS expression in mice

**Fig 4:**
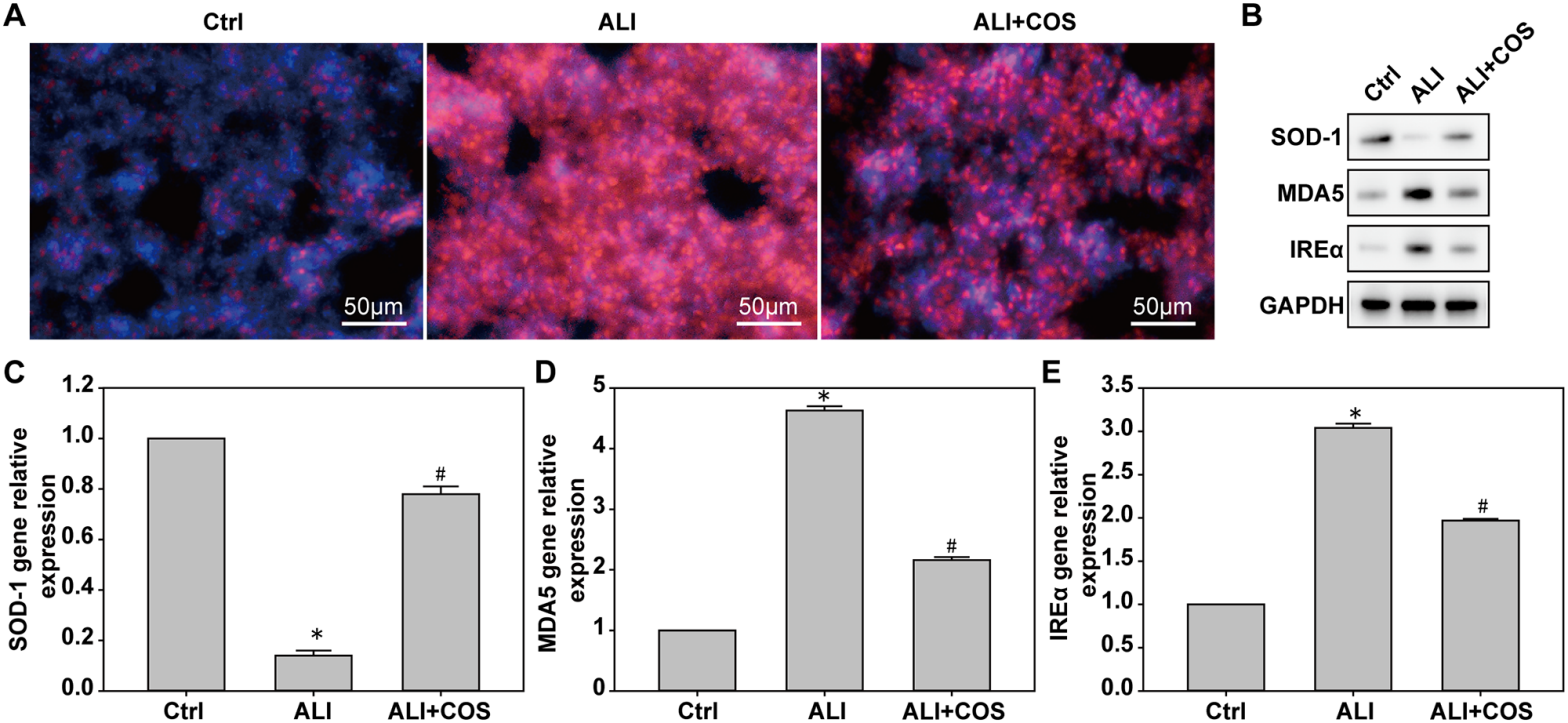
COS effects on oxidative stress in the lung. Oxidative stress in the lung was determined using immunofluorescence staining, real time-PCR and western blot. *P < 0.05, vs. control group; ^#^*P* < 0.05, vs. ALI group. COS: chitosan oligosaccharide; ALI: acute lung injury.

Compared with the control group, ROS expression was significantly higher in the ALI group. Compared with the ALI group, ALI-induced ROS expression was significantly suppressed in the ALI + COS group (Fig 4A, P < 0.05).

### COS alleviates blast injury-induced oxidative stress in mice

Compared with the control group, the expression of MDA5 and IREα mRNA and proteins were significantly decreased, while SOD-1 mRNA and protein expression was significantly increased in the ALI group. Compared with the ALL group, the expressions of MDA5 and IREα mRNA and protein were significantly increased, while SOD-1 mRNA and protein expression was significantly decreased in the ALI + COS group (Fig 4B–4E, P < 0.05).

### COS decreases blast injury-induced apoptosis in mice

**Fig 5:**
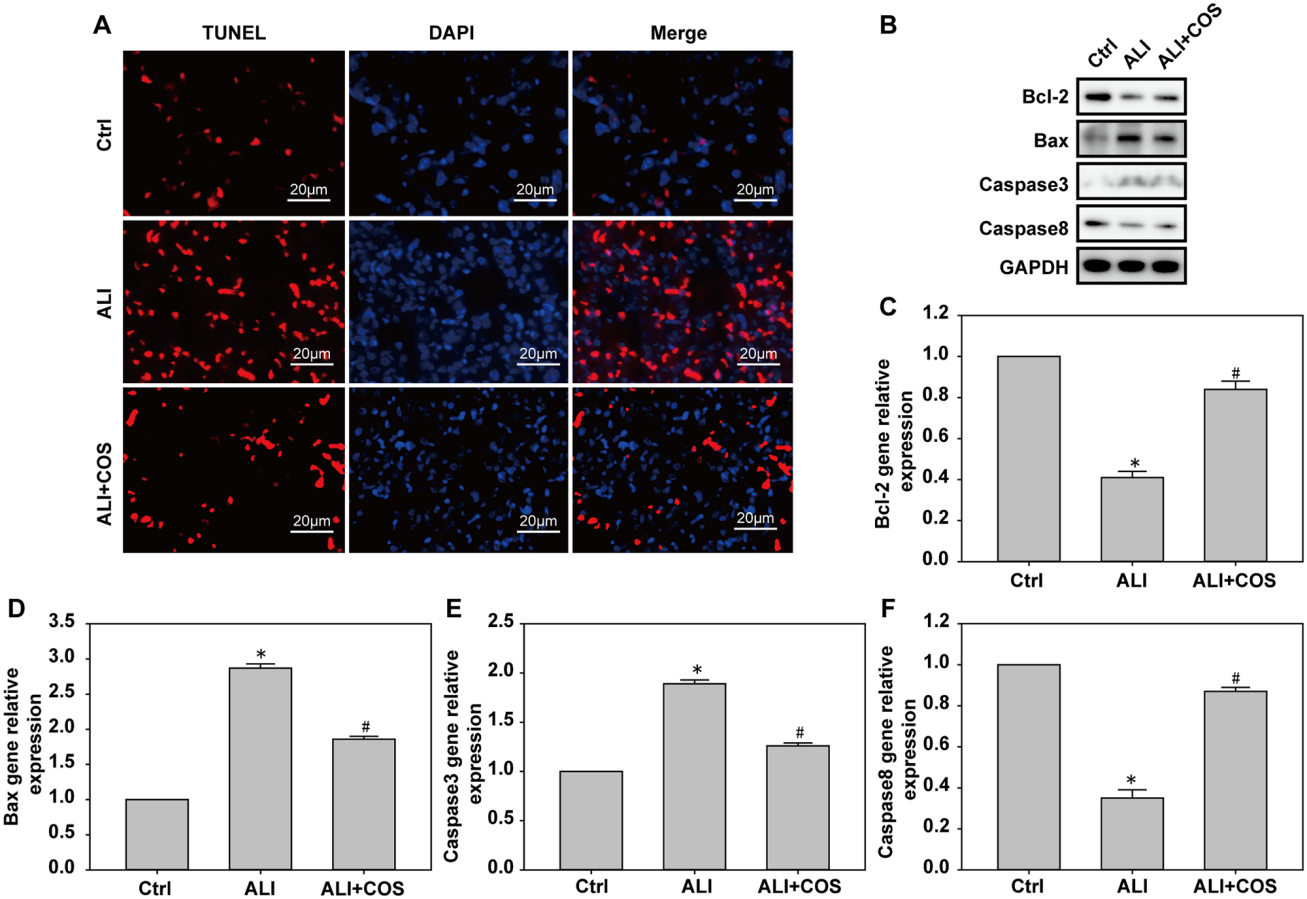
COS effects on apoptotic protein expression in the lung. Apoptotic protein expression in the lung was detected using TUNEL, real time-PCR and western blot. *P < 0.05, vs. control group; ^#^*P* < 0.05, vs. ALI group. COS: chitosan oligosaccharide; ALI: acute lung injury; TUNEL: terminal deoxynucleotidyl transferase dUTP nick end labeling.

Compared with the control group, the number of apoptotic cells was significantly increased in the ALL group. In contrast, COS significantly reduced the number of apoptotic cells (Fig 5A). Compared with the control group, the expressions of Bax and Caspase-3 mRNA and protein were significantly increased, while Bcl-2 and Caspase-8 mRNA and proteins expression were significantly decreased in the ALI group. Compared with the ALI group, the expression of Bax and Caspase-3 mRNA and protein were significantly decreased, while Bcl-2 and Caspase-8 mRNA and protein expresions were increased in the ALI + COS group (Fig 5B–5F, *P* < 0.05).

### COS ameliorates blast injury-induced ALI through DDAH1-/-ADMA / p38 signaling in mice

**Fig 6:**
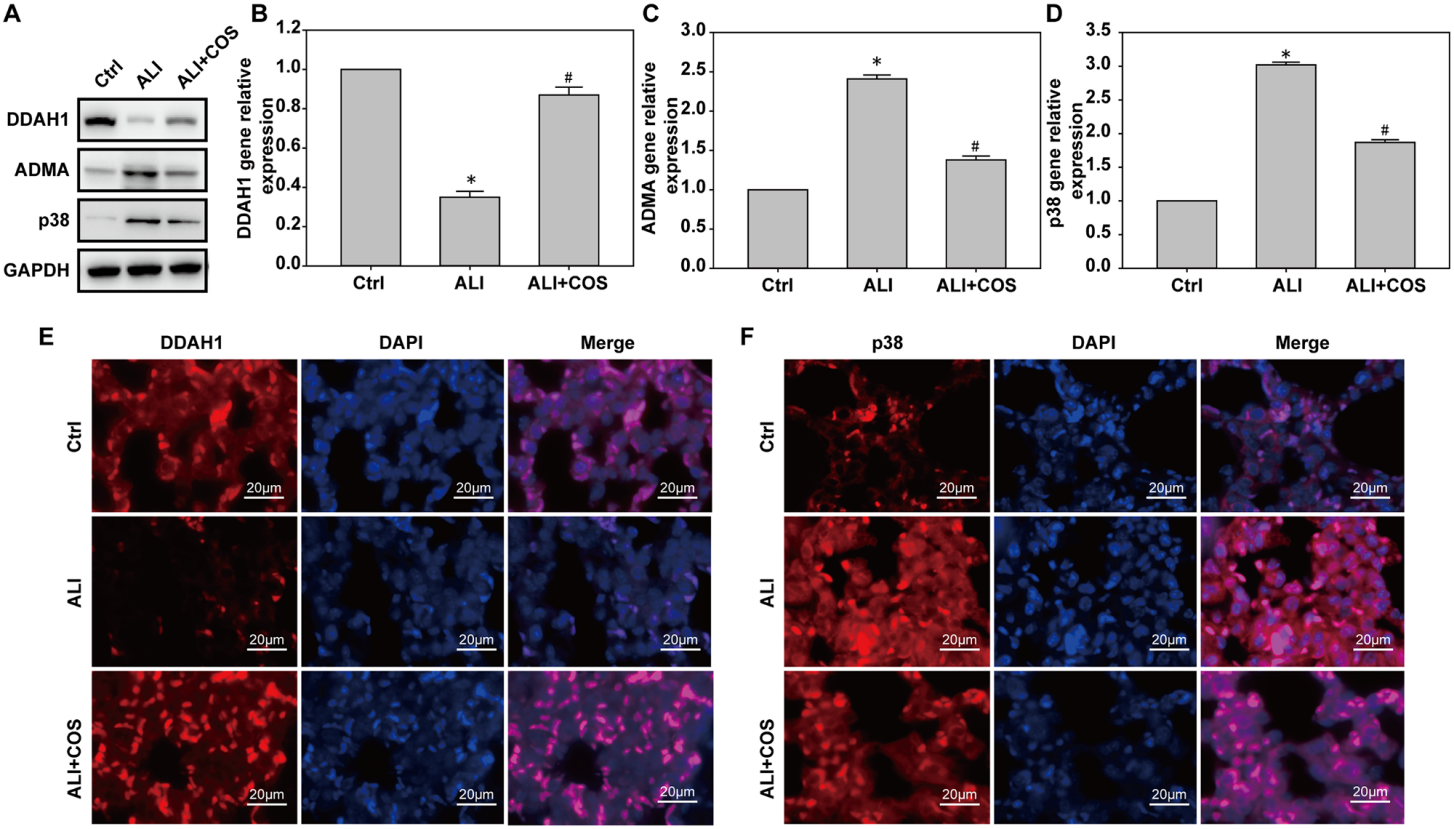
COS effects on pathway protein expression in the lung. Pathway protein expression in the lung was measured using immunofluorescence staining, real time-PCR and western blot. *P < 0.05, vs. control group; ^#^*P* < 0.05, vs. ALI group. COS: chitosan oligosaccharide; ALI: acute lung injury.

Compared with the control group, mice in the ALI group had lower DDAH1 expression and higher p38 expression. COS significantly increased the expression of DDAH1, and reduced p38 expression((Fig 6A and 6B). Compared with the control group, DDAH1 mRNA and protein expressions were decreased, and ADMA and p38 mRNA and protein expressions were significantly increased in the ALI group. Compared with the ALI group, DDAH1 mRNA and protein expression were increased, while ADMA and p38 mRNA and protein expression were decreased in the ALI + COS group (Fig 6B–6E, *P* < 0.05)

## Discussion

COS and its derivatives have various biological activities, including anti-inflammation, immunostimulation, anti-tumor, anti-obesity, anti-hypertension, anti-microorganism and anti-oxidation actions, and protect against Alzheimer’s disease [20]. This study confirmed that COS mitigated blast injury-induced ALI, inhibited the inflammatory reaction, and reduced oxidative stress and cell apoptosis. Moreover, COS altered inflammation-related pathway protein expression after blast injury-induced ALI. Our results suggest that COS mitigates blast injury-induced ALI through the DDAH1/ADMA pathway.

In this study, COS markedly improved blast injury-induced ALI, inhibited NF-κB, TNF-α, IL-1β, IL-4 and IL-6 expression, and promoted IL-10 expressions. In previous studies, Kunanusornchai et al.[21] found that oral administration of COS (10 mg/kg/day) induced AMP-activated protein kinase activation, and suppressed TNF-α-induced iNOS and COX-2 expression in rabbit and human synovitis cells, indicating that COS suppressed synovitis through AMP-activated protein kinase activation. Huang et al.[22] demonstrated serum TNF-α, IL-6,IL-8,p-NF-κB p65, IKKα/β and IκB protein expression were reduced, and anti-inflammatory cytokine expression was significantly increased in piglets fed with COS. These findings suggested that COS diminished the intestinal inflammatory reaction, and that its mechanism of action might be associated with inhibition of the NF-κB signaling pathway. A previous study[23] showed that COS reduced LPS-induced organ dysfunction, reduced serum TNF-α and IL-1β expressions, and improved the imbalance of glutathione, catalase and malondialdehyde levels. Therefore, COS has a significant protective effect on blast injury-induced inflammatory reactions, and plays an anti-inflammatory role by suppressing the expression of proinflammatory cytokines and elevating the expression of antiinflammatory cytokines.

In the present study, blast injury caused increased MDA5 and IREα expresions, inhibited SOD-1 expression resulting in oxidative stress, increased Bax and Caspase-3 protein expression, suppressed Bcl-2 and Caspase-8 protein expression, leading to cellular apoptosis. SOD-1, a number of the SOD family, is a natural scavenger of oxygen free radicals, and improves lipid peroxidation caused by superoxide anion radical, cell membrane damage, inflammation, tumors and autoimmune disease[24, 25]. Caspase family members have an important role in apoptotic signal transduction of the endoplasmic reticulum, and activated caspases induce cell apoptosis. Caspase-3 is a key enzyme that mediates apoptosis [26]. Activated caspase-3 degrades Bcl-2 protein to prevent its anti-apoptotic effect,however, once caspase-3 is activated, the occurrence of apoptosisis irreversible[27]. Bax is a pro-apoptotic protein that is transfer from the cytoplasm to the mitochondria after apoptosis, causing cytochrome C release and apoptosis[28]. We found that COS significantly inhibited blast injury-induced MDA5 and IREα expresions, and promoted SOD-1 expression. Simultaneously, COS markedly suppressed Bax and Caspase-3 protein expressions, enhanced Bcl-2 protein expression, and increased nonactivated Caspase-8 expression. Jia et al.[29] demonstrated that the oral administration of COS reduced learning and memory deficits induced by Aβ1–42, improved neuronal apoptosis, diminished malondialdehyde and 8-hydroxy-2’-deoxyguanosine levels, and increased the activity of glutathione peroxidase and superoxide dismutase in rats, indicating that the neuroprotective effect of COS was strongly associated with its ability to inhibit oxidative stress. Huang et al.[30] found that COS downregulated Cu(2+)-induced oxidative stress in SH-SY5Y cells, activated Caspase-3, and increased the expression levels of pSer40-Nrf2 and HO-1 proteins. Their results confirm that COS may play a protective role in Cu(2+)-induced oxidative damage by activating Nrf2 protein. Moreover, a previous study[31] verified that COS inhibited the increase of intracellular ROS induced by Cu(2+). These studies indicate that COS may have a protective role against lung blast injury by improving oxidative stress and apoptosis induced by blast injury.

The protective mechanism of COS involves several important pathways, including the inhibition of NF-κB, mitogen-activated protein kinase (MAPK) and AMP-activated protein kinase[21]. p38 is a mitogen activated protein kinase involved in cell differentiation, inflammation, apoptosis and autophagy[32, 33]. p38 MAPK was activated in patients with trauma, accompanied by increased levels of TNF-α and IL-6, and their expressions were positively associated with the degree of trauma. TNF-α and IL-1 activate p38 pathway, and produce a cascade effect[34]. ADMA and DDAH1 are of increasing interest in the fields of heart and lung research, and are associated with asthma, shock and cancer[35]. ADMA is mainly metabolized through DDAH1 and DDAH2, and a low L-arginine/ADMA ratio is associated with decreased pulmonary function[36]. In coronary endothelial cells, ADMA-activated p38 MAPK and ADMA-induced upregulation of ACE were inhibited by p38 MAPK, and caused a prolong increase in plasma ADMA and cardiac oxidative stress, vascular injury. These results indicate that the DDAH2/ADMA pathway may be a novel therapeutic target for ADMA- or angiotensin II-induced vasculopathy[37]. In the current study, blast injury decreased DDAH1 expression and an increased ADMA and p38 expressions in the lung. COS markedly increased DDAH1 expression and inhibited ADMA and p38 expression. Li et al.[38] investigated the effects of ADMA/DDAH1 pathways on fatty degeneration of the liver induced by a high-fat diet in DDAH1 knockout mice, and found that deletion of the DDAH1 gene induced oxidative stress, endoplasmic reticulum stress and inflammatory reactions in hepatocytes. Moreover, the over-expression of DDAH1 reduced palmitic acid-induced fatty degeneration, oxidative stress and inflammation. A previous study showed that the expressions of DDAH1 and DDAH2 in the lungs of DDAH1 transgenic mice were decreased by intratracheal attack of PBS or house dust mite and were associated with elevated levels of ADMA in the serum and bronchoalveolar lavage fluid. These results suggest that the reduced expression of DDAH1 and DDAH2 in the lung may be induced by allergen-induced airway inflammation[39]. As described above, COS mitigated blast injury-induced inflammatory reactions, protected pulmonary functions, and mitigated lung injury probably through the DDAH1/ADMA/p38 cell signaling pathway.

In summary, blast injury leads to inflammation, oxidative stress and apoptosis in the mouse lung. COS has a protective effect on blast injury-induced lung injury possibly through promoting DDAH1 expression and suppressing the ADMA and MAPK pathways.

## Supporting information

S1 File COS effects on organ weight(raw data). (XLSX)
